# Perspective on Similarities and Possible Overlaps of Congenital Disease Formation—Exemplified on a Case of Congenital Diaphragmatic Hernia and Neuroblastoma in a Neonate

**DOI:** 10.3390/children8020163

**Published:** 2021-02-22

**Authors:** Zihe Huo, Remo Bilang, Benedikt Brantner, Nicolas von der Weid, Stefan G. Holland-Cunz, Stephanie J. Gros

**Affiliations:** 1 Department of Pediatric Surgery, University Children’s Hospital Basel, 4031 Basel, Switzerland; zihe.huo@ukbb.ch (Z.H.); remo.bilang@ukbb.ch (R.B.); benedikt_brantner@hotmail.com (B.B.); Stefan.Holland-Cunz@ukbb.ch (S.G.H.-C.); 2 Department of Clinical Research, University of Basel, 4001 Basel, Switzerland; nicolas.vonderweis@ukbb.ch; 3 Department of Hematology and Oncology, University Children’s Hospital Basel, 4056 Basel, Switzerland

**Keywords:** CDH, differentiation, hypoxia, neuroblastoma, retinoic acid pathway, rare disease, orphan disease, pediatric surgery, NMYC, AQP1

## Abstract

The coincidence of two rare diseases such as congenital diaphragmatic hernia (CDH) and neuroblastoma is exceptional. With an incidence of around 2–3:10,000 and 1:8000 for either disease occurring on its own, the chance of simultaneous presentation of both pathologies at birth is extremely low. Unfortunately, the underlying processes leading to congenital malformation and neonatal tumors are not yet thoroughly understood. There are several hypotheses revolving around the formation of CDH and neuroblastoma. The aim of our study was to put the respective hypotheses of disease formation as well as known factors in this process into perspective regarding their similarities and possible overlaps of congenital disease formation. We present the joint occurrence of these two rare diseases based on a patient presentation and immunochemical prognostic marker evaluation. The aim of this manuscript is to elucidate possible similarities in the pathogeneses of both disease entities. Discussed are the role of toxins, cell differentiation, the influence of retinoic acid and NMYC as well as of hypoxia. The detailed discussion reveals that some of the proposed pathophysiological mechanisms of both malformations have common aspects. Especially disturbances of the retinoic acid pathway and NMYC expression can influence and disrupt cell differentiation in either disease. Due to the rarity of both diseases, interdisciplinary efforts and multi-center studies are needed to investigate the reasons for congenital malformations and their interlinkage with neonatal tumor disease.

## 1. Introduction

Rare diseases are defined by their relative severity and their low prevalence. Depending on the country or region, the definition of a rare disease ranges between a prevalence of lower than 0.1–7.5 per 10,000 inhabitants [1,2,3]. About 7000 rare diseases and disorders have been identified [3]. Roughly 50 million patients in Europe and the US are diagnosed with rare diseases. Half of these patients are children [2]. The coincidence of two rare diseases is, therefore, exceptional, but can occur in children and neonates. Oftentimes, the causes of rare congenital diseases are not completely understood yet.

Congenital diaphragmatic hernia (CDH) is a rare condition in neonates with a prevalence of 2.3 in 10,000 births [4]. Any disturbance in the pleuroperitoneal membranes before the 12th week of gestation may result in a diaphragmatic defect. Anatomically, the most common hernia is located at the postero-lateral part of the diaphragm (70% to 75%, Bochdalek triangle, lumbocostal trigonum), and a majority of the hernias occur on the left side. Most cases nowadays are diagnosed in utero due to improvements in prenatal imaging. Improvements in surgery as well as supportive measures have led to an overall survival rate ranging between 60% and 70% for CDH [5]. In around 30% of cases, CDH is associated with other anomalies, most commonly cardiac anomalies followed by anomalies of the urinary tract, limbs and the nervous system [6]. Hypotheses regarding the pathogenesis of CDH include interruption of the formation of the diaphragm by fusion of the septum transversum anteriorly, the pleuroperitoneal folds dorsolaterally, the crura from the esophageal mesentery dorsally and the body wall mesoderm posteriorly [7,8]. Another hypothesis revolves around the failure of muscularization of the developing diaphragm prior to complete closure of the pleuroperitoneal canal [9].

Neuroblastoma is one of the most common solid malignancies in children; nevertheless, it is a rare disease with an incidence of 1:7000 to 1:8000 live births. Neuroblastoma contributes to 15% of child deaths from cancer [10]. However, the prognosis strongly depends on the risk profile of the tumor. Neuroblastoma is classified into four risk categories by the International Neuroblastoma Risk Group (INRG) classification system, which is based on several factors including age at diagnosis, tumor stage, histology, differentiation grade, copy number status of NMYC and deletion status of chromosome 11q among other segmental chromosomal abnormalities [11]. Neuroblastoma primary tumor and metastases are biologically very heterogeneous. They can present with clinical manifestations ranging from spontaneous regression to highly aggressive metastatic disease that is unresponsive to standard and investigational anti-cancer treatments [12]. Neuroblastoma most often develops in the adrenal medulla or other locations originating from the neural crest, such as the paraspinal sympathetic ganglia or the pelvic ganglia. Several genes that are involved in the regulation of neural crest development are expressed in neuroblastoma as well [13]. Neuroblastoma cells can present in varying differentiation stages, with heterogeneous phenotypes ranging from an initial epithelial-like one to a more branched and neuronal one [14]. How migrating neural crest cells convert to sympathetic precursors and, finally, to differentiated sympathetic neuronal cells has not yet been completely understood. Interruptions of this process, however, are made accountable for the formation of neuroblastoma.

Established and experimental adverse prognostic factors of neuroblastoma include amplification of NMYC, expression of the chemokine receptor CXCR4 that is responsible for metastatic homing and the hypoxia-dependent water channel aquaporin 1 (AQP1). NMYC is essential for the differentiation state of neuroblastoma cells and the fate of neural crest cells [15,16]. Knockdown of NMYC leads to apoptosis and terminal differentiation of the cells [17]. The chemokine receptor CXCR4 belongs to a large family of cytokine receptors. It is expressed in various cancers and is, therefore, well studied [18]. It has been shown that high expression of CXCR4 in neuroblastoma correlates with a significantly impaired outcome compared to low CXCR4 expression [19,20]. Expression of the water channel aquaporin 1 (AQP1) has been linked to a hypoxic cell phenotype with a higher expression of hypoxia-inducible factor (HIF)-1α and HIF-2α [21] (Huo et al., published February 2021, *Front Cell Dev Biol*.). This goes hand in hand with a greater potential of AQP1-positive cells to migrate in neuroblastoma.

The aim of our study was to put the respective hypotheses of disease formation of CDH and neuroblastoma as well as known factors in this process into perspective regarding their similarities and possible overlaps in congenital disease formation.

## 2. Materials and Methods

### 2.1. Tumor Tissue

Patient tumor samples resected in the course of regular treatment and which were not needed for clinical investigations were used after patient consent, as approved by the appropriate ethics committee (Ethikkommission Nordwest- und Zentralschweiz, EKNZ 2015-263). Tumor tissue was taken from the resected specimen immediately after surgery and snap-frozen or placed in formalin and embedded in paraffin.

### 2.2. H&E and Immunostaining

Next to routine pathological analysis, paraffin sections were stained by standard H&E (Hematoxylin and Eosin) and immunohistochemical staining for experiments. Cryosections were used for immunofluorescence staining.

Immunohistochemistry staining was performed using the HRP-AEC-System from R&D Systems (Biotechnology Company, Minneapolis, MN, USA) with polyclonal rabbit anti-AQP1 antibody (Merck Millipore, Darmstast, Germany) at a dilution of 1:400 and counterstaining was performed with Mayer’s hematoxylin solution (Spitalpharmazie, Basel, Switzerland). As a control, sections were incubated with antibody diluent (DAKO, Glostrup, Denmark) without primary antibody at 4 °C overnight and then treated as other samples.

For AQP1, MAP2 (microtubule associated protein) and CXCR4 immunofluorescence staining, the polyclonal rabbit anti-AQP1 antibody (Merck Millipore, Darmstadt, Germany), the monoclonal mouse MAP2 antibody (Abcam, Cambridge, UK) and the polyclonal goat CXCR4 antibody (Abcam, Cambridge, UK) were used at dilutions of 1:400, 1:200 and 1:100, respectively, following a standardized protocol. As secondary antibodies, goat anti-mouse IgG1 AlexaFluor 488 and goat anti-rabbit AlexaFluor 647 were used. Negative controls missing the primary antibody were included in every staining cycle. Slides were mounted using a ProLongVR Gold Antifade Mountant with DAPI (4′,6-diamidin-2-phenylindol) (Life Technologies, Thermo Fisher Scientific, Waltham, MA, USA). Slides were analyzed with an Olympus BX43 microscope using CellSens software.

## 3. Results

We present the joint occurrence of two very rare diseases based on a patient presentation and immunochemical prognostic marker evaluation.

**Figure 1:**
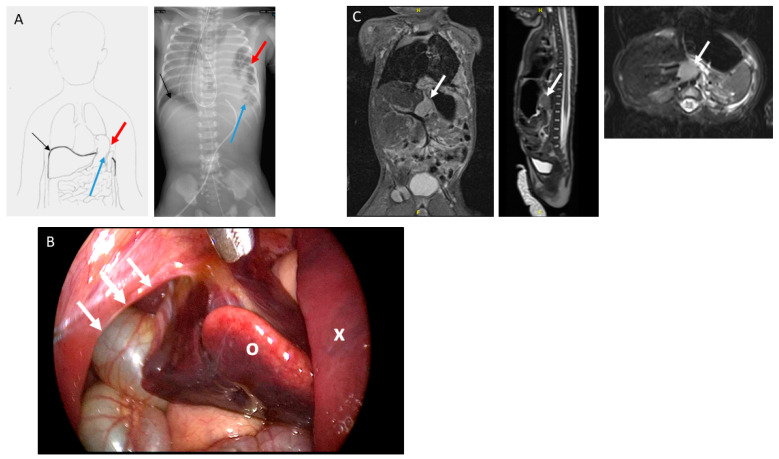
Diagnosis of congenital diaphragmatic hernia (CDH) and neuroblastoma. Intraoperative situs at hernia repair. (**A**) Anatomical conditions in the patient regarding the congenital diaphragmatic hernia schematically (left panel) and on patient X-ray (right panel). Black arrow—regular right-sided diaphragm; blue arrow—left-sided diaphragmatic defect; red arrow—intrathoracic intestine. (**B**) The intraoperative situs at time of hernia repair in shown case. The edge of the diaphragm is marked by white arrows, the left lung is indicated by x and the metastatic liver segment reaching through the diaphragmatic defect by O. (**C**) Magnetic resonance imaging (MRI) shows the neuroblastoma retroperitoneally behind the liver and stomach in front of the abdominal aorta and inferior vena cava (left panel—coronal view; middle panel—sagittal view; right panel—transversal view). Close proximity is given to the liver and the great vessels. In each panel, the primary tumor is indicated by an arrow. MRI was performed after hernia repair.

**Figure 2:**
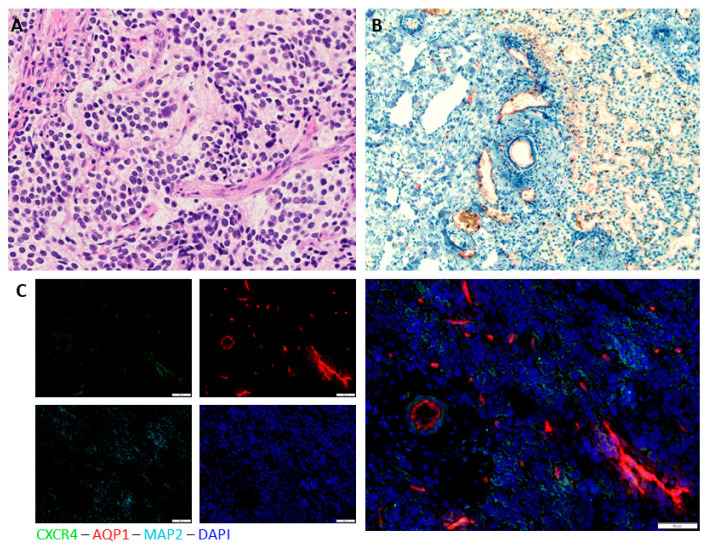
H&E (Hematoxylin and Eosin) and immunohistochemical staining of primary tumor and metastases. (**A**) H&E staining of primary tumor shows histomorphological characteristics of poorly differentiated, Schwannian stroma poor neuroblastoma; (**B**) aquaporin 1 (AQP1) immunohistochemical staining and hematoxylin counterstaining of the liver metastases reveals dissolution of the periportal structure in the tumor-infiltrated regions. AQP1 positivity is observed in metastatic areas as well as in vascular endothelium (which can serve as an internal positive control). (**C**) AQP1, CXCR4 and MAP2 (microtubule associated protein) immunofluorescence staining of the primary tumor. Nuclei staining with DAPI (4′,6-diamidin-2-phenylindol). Next to AQP1, which is expressed in vascular structures as well as specific tumor areas, there is some expression of CXCR4 as a marker for metastatic homing as well as MAP2 expression in some areas indicating a more mature neuronal phenotype. Overall, tumor and metastases express experimental features that can be associated with a more aggressive phenotype.

The female patient was born by primary caesarean section with a weight of 2490 g and an APGAR (Appearance, Pulse, Grimace, Activity and Respiration) score of 3/6/6 at 38 + 3 weeks of gestation. The mother had been treated for hyperemesis gravidarum in the first trimester and mild hypertension during the course of pregnancy. Maternal serologies as well as medical history were inconspicuous. No other complications were reported until a routine ultrasound in the third trimester reported a suspicion of a congenital diaphragmatic hernia on the left side. MRI was performed during the 28th week of gestation, which confirmed a CDH with herniation of the intestines, the gut and the spleen through the left thoracic aperture. A lung-to-head ratio (LHR) of 2.3 and a lung volume on the left side of 2.5 mL were calculated. After further initial neonatal and cardiological evaluations, a thoracoscopic diaphragmatic hernia repair was attempted on the sixth day of life. The colon, small intestine and spleen were herniating into the thoracic cavity (Figure 1A). Furthermore, a mass resembling a lung sequester or an altered part of the liver was found that presented in close anatomical proximity to the aorta (Figure 2B). The mass was resected via a laparotomy. Hernia closure was achieved with a Gore-Tex^®^ patch. Further intraoperative findings include the malrotation of the intestine.

A histopathological examination of the resected mass revealed liver metastases of a neuroblastoma (Table 1). Consecutively, urine analysis showed an increased vanillylmandelic acid (VMA)-to-creatinine ratio of 13.5 µmol/mmol (ref. < 11) and a normal homovallinic acid (HVA)-to-creatinine ratio of 10 µmol/mmol (ref. < 20). Post-operatively conducted magnetic resonance imaging (MRI) could distinguish a 2 × 1.4 × 1.4-cm retroperitoneal mass between the aorta and the inferior vena cava (Figure 1C). The MIBG (metaiodbenzylguanidin) scintigraphy showed a moderate enrichment of the described mass as well as multiple metastases in the liver. No signal enhancement was found in the bones and examination of the bone marrow was inconspicuous. Due to problems of the gastrointestinal passage, a re-laparotomy was performed. Adhesiolysis and a Nissen fundoplication were performed and the passage was restored. During this operation, a biopsy of the primary tumor was taken. In consideration of the immunohistochemical examination and the fluorescence in situ hybridization (Table 1) as well as the age of the patient and the results of the first pathological findings on the liver metastases, the tumor was defined as peripheral neuroblastoma, stage IV. The CGH array (comparative genomic hybridization) performed on the liver metastases at diagnosis showed no SCA (segmental chromosomal aberrations); therefore, the child received no immediate chemotherapy. The interdisciplinary tumor board agreed to start watchful waiting. The postoperative course was uneventful, without signs of recurrence, chylothorax or gastroesophageal reflux. The patient recovered well and consecutively showed sufficient weight gain.

Seven months later, a progression of the primary tumor was reported on MRI. The case was re-discussed by the interdisciplinary tumor board and the decision was made to retrieve a further biopsy of the primary tumor to rule out biological tumor changes. A CGH array was repeated on the primary tumor tissue and showed SCA (1p deletion). No immunohistochemical changes of the tumor were found. The key findings of the analysis of the biopsies are summarized in Table 1. On account of the progression and SCA, chemotherapy was indicated. According to the SIOPEN (International Society of Paediatric Oncology—Europe-Neurobalstoma/Low and Intermediate risk NEuroblaStoma)/LINES (protocol, a four-cycle chemotherapy with VP-16 (etoposide) and carboplatin was initiated). The chemotherapy was well tolerated and the patient showed a partial remission. At this time, surgical resection of the primary tumor was not an option and two further cycles of CADO (cyclophosphamide, doxorubicin, and vincristine) were initiated. The re-evaluation showed a very good partial remission with a primary tumor <1 cm and no changes in the metastases. Due to the positive response, the girl is now under a close follow-up setting. Regarding lung development and respiratory function, she is developing very well. There are no clinical respiratory restrictions.

### Immunohistochemical Analysis

The primary tumor showed histopathological characteristics of a poorly differentiated, Schwann cell-poor stroma with a proliferation rate (MIB1, E3 ubiquitin-protein ligase) of 40%, a low mitosis karyorrhexis index (INPC, International Neuroblastoma Pathology Classification) and <5% differentiating cells. The histological prognosis according to INPC is favorable. NMYC is not amplified and the tumor is synaptophysin-, chromogranin A- and neurofilament-positive.

AQP1 staining and hematoxylin counterstaining of the liver metastasis showed dissolution of the periportal structure in the tumor-infiltrated regions (Figure 2A). AQP1 positivity is observed in metastatic areas as well as in the vascular endothelium, in which it is expected to be physiologically positive (Figure 2B). Expression of AQP1 in metastatic cells could point to a more aggressive, metastatic cell phenotype in the metastatic cells, as demonstrated earlier by our group [21] (Huo et al., published February 2021, *Front Cell Dev Biol.*).

Immunofluorescence staining of AQP1, CXCR4 and MAP2 of the primary tumor revealed AQP1 expression in some but not all tumor areas, as well as staining of vascular structures in which AQP1 occurs physiologically (Figure 2C). Expression of CXCR4 as a marker for metastatic homing cannot be widely observed but is present in some tumor cells. MAP2 expression in some tumor areas could indicate a more mature neuronal phenotype. Despite the routine histological good prognosis, the tumor and metastases express features that have previously been associated with a more aggressive phenotype of neuroblastoma [21] (Huo et al., published February 2021, *Front Cell Dev Biol*.). This could explain the clinical progression and eventual need for chemotherapy.

## 4. Discussion

There are no explicit data connecting the occurrence of CDH with neuroblastoma in a neonate. Moreover, both diseases are classified as rare. Nonetheless, there are some hints in the literature that could indicate a common causality of these two disease entities. It has, for example, been shown that space-occupying lesions such as bronchopulmonary sequestration, ectopic liver and foregut duplication cysts are common pathological findings in neonates with congenital diaphragmatic hernia [22]. Furthermore, it has been described that children with congenital malformations have an increased risk for various malignancies [23]. It has also been suggested that neonatal tumors are more often associated with congenital abnormalities than other pediatric cancers [24].

**Figure 3:**
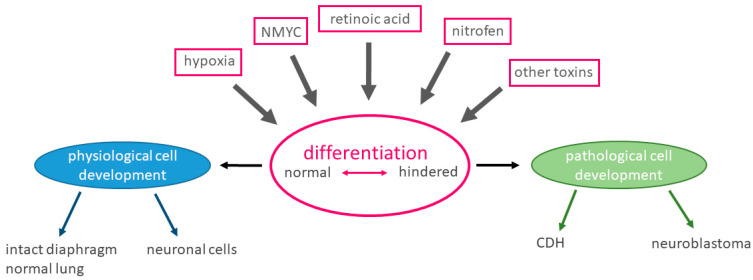
Schematic view of hypothesis: Possible influences on differentiation. The schematic view shows possible influences on physiological differentiation and development, including both possible pro-differentiation influences such as retinoic acid and NMYC as well as anti-differentiation influences such as nitrofen, other toxins and hypoxia. Timing and interaction of the physiologically occurring influences is essential in this developmental process. We hypothesize that a faulty differentiation at a specific time in development or recurring exposure to toxins can lead to the simultaneous occurrence of both CDH (congenital diaphragmatic hernia) and neuroblastoma. They might even arise from the same cause.

Could the simultaneous occurrence of a neonatal tumor and a congenital malformation be connected by a common mechanism during embryological development? We will discuss aspects of embryogenesis, localization, retinoic acid-induced differentiation, NMYC-driven differentiation and disturbance of physiological processes by hypoxia as possible common mechanisms (Figure 3).

### 4.1. Embryogenesis

Normal development of the diaphragm occurs during the fifth and seventh week of gestation by the fusion of the mesenchymal septum transversum and the pleuroperitoneal folds (PPF) [25]. Key factors for the proper fusion of the PPF are the muscle progenitors which migrate from the somites into the PPF as well as the projection of the phrenic nerve from the C3–C5 segment into the PPF [26,27]. In approximately 30% of CDH cases, genetic causes can be identified. Today, a wide variety of genetic defects are known. Among these are disturbances of the retinoic acid pathway [28], which, in turn, has been shown to play a role in the differentiation of neuroblastoma. One of the most commonly used models to induce CDH in animal experiments is utilization of the teratogen nitrofen. It has been shown that between day 7 and day 20 of gestation in a rat model, the embryo undergoes an increased retinol metabolism and thus has an increased need for vitamin A during this time period [29]. Neuroblastoma, on the other hand, arises from the neural crest. Due to a not-yet fully understood mechanism, neuronal progenitor cells proliferate and permanently adapt a mostly undifferentiated phenotype. Vitamin A efficiently inhibits proliferation of neuroblastoma cells and leads to neuronal differentiation [30,31,32,33]. As 13-cis-retinoic acid, it is used as a maintenance therapy in high-risk neuroblastoma patients with minimal residual disease [34]. There are, however, no sufficient data on the hypothesis that, reciprocally, a lack of retinoic acid increases the risk of neuroblastoma development.

### 4.2. Localization

The primary tumor of our patient was located retroperitoneally in the space between the aorta and the inferior vena cava. The resected liver part was also found in close proximity to the aorta. It could, thus, be hypothesized that tumor development at this specific location could present a mechanical disturbance of the diaphragm during development. As another possible reason for mechanical disturbance, liver heterotopia can be found in the literature, although the coincidence of impaired diaphragm development and liver heterotopia is a rarely described condition [22]. Cruz et al. investigated the outcome of CDH with space-occupying lesions (SOLs) and enrolled 214 participants diagnosed with CDH. Of all the participants, nine were reported to have a liver heterotopia [22]. In a literature review published in 2019, Mito et al. reported no more than 19 cases of liver heterotopia associated with CDH. It is noteworthy that of the 19 described cases, none coincided with tumorization [25]. Neither article discussed an embryogenetic connection of CDH and ectopic liver.

The endodermal liver bud first appears three weeks after gestation and bone morphogenetic proteins released by the septum transversum lead to development of the liver [25]. An important chemo-attractant which determines the direction of liver development appears to be neurturin (NRTN), which is expressed by the ductus venosus. Ectopic production of NRTN may lead to ectopic liver development. However, a correlation to CDH has not been described [35].

The close anatomical proximity of the hepatobiliary system and the diaphragm leads to the hypothesis that a disturbance in either of the organs affects the development of the other. However, there is no explicit research regarding the mechanical disturbance in the embryogenesis of the diaphragm. In view of the unrelated time point of development, a correlation between these two occurrences seems unlikely but is not excluded regarding the current level of knowledge.

### 4.3. Retinoic Acid Pathway

Among the possible causes of CDH, disturbances of the retinoic acid pathway have been described [28], while retinoic acid is essential for neuronal differentiation. We hypothesize that a faulty retinoic acid pathway could be responsible for both diseases.

Retinoic acid as well as vitamin A play key roles in early physiological embryogenesis and organ development [36,37,38]. Disruption of the retinoic acid pathway has been shown to lead to faulty embryonic development and organ differentiation. It has also been shown to have a major effect on the differentiation of neuroblastoma cells [14]. It has thus been hypothesized that disruption of the retinoid acid pathway contributes to malformation of the diaphragm. There are several findings endorsing this theory. Early on, Anderson and his group found an increased incidence of CDH in young rats that underwent a vitamin A-deficient diet [39]. Experiments in which rats were supplemented with vitamin A during gestation showed a decreased incidence of CDH [39]. It has been shown that retinoid receptors are expressed on the developing diaphragm [40]. Retinoid receptor-null mutant mice lacking retinoid acid receptors showed an increased incidence of CDH.

Similarities were observed between nitrofen-induced diaphragmatic hernia formation and malformations arising from disturbance of the retinoic acid pathway [41]. An interference with the synthesis of retinoid acid by nitrofen can be assumed based on these data [41]. An explanation of the severe effect of nitrofen on the development of the diaphragm in these models may lay in the increased retinol metabolism, with several peaks of vitamin A accumulation between day seven and day twenty of gestation [29]. Thus, a higher susceptibility to changes in the retinoic pathway during this period seems possible [41]. This could be relevant for neuronal development and differentiation.

No link between neuroblastoma cell differentiation and CDH formation in the context of the retinoic acid pathway has been described until now. From the evidence discussed above, it seems, however, possible that disturbances in the retinoic acid pathway may result in both congential diaphragmatic hernia and neuroblastoma.

### 4.4. Influence of NMYC

NMYC protein expression in humans and in mice occurs physiologically in specific tissues at specific embryonic developmental stages and plays a pivotal role in embryonic development [42]. It is mostly found in tissues of the developing kidney, intestine, lung and heart [43,44]. Mutations of NMYC have been linked to these defects. NMYC expression is very low or absent in most adult tissues [43,44,45,46,47,48,49]. NMYC knockout mice die around E22.5 and display hypoplasia of different organs and tissues, including an underdeveloped central and peripheral nervous system and defects in lung branching and in the morphogenesis of the genitourinary system, stomach, intestine and limb buds [44,50,51,52,53,54]. NMYC induces the expression of several pluripotent genes in neuroblastoma and neuronal progenitor cells [55]. A knockdown of NMYC in neuronal tissues leads to an expansion of neuronal progenitor cells [56]. It has been suggested that NMYC function plays a vital role in controlling early differentiation in several tissues, including neuronal cells [47,49]. This has been supported by the finding that NMYC overexpression in the avian neural crest as well as in the murine neural crest leads to an increased generation of neurons [16,57]. In both studies, a downregulation of NMYC was suggested to be necessary for terminal differentiation of neurons.

However, the retinoic acid-induced differentiation in the context of neuroblastoma has been shown to be independent of NMYC [30,31,32,33,58,59]. Interestingly, there is evidence that the downregulation of NMYC precedes the retinoic acid-induced differentiation. It has, furthermore, been suggested that retinoic acid can directly regulate NMYC at the transcriptional level [32]. It has been shown that a knockdown of NMYC in neuroblastoma leads to morphological and biochemical neuronal differentiation [60,61,62]. Thus, NMYC may play a major role in maintaining an undifferentiated phenotype in neuroblastoma [15].

NMYC, therefore, can be considered as another key player in differentiation. Furthermore, in SH-EP Tet-21/N cells that were treated with tetracycline and, thus, do not amplify NMYC, we found that although NMYC is downregulated, several other adverse factors such as AQP1, HIF-1α and HIF-2α are upregulated [21].

### 4.5. Role of Hypoxia and Hypoxia-Associated Factors in Development

Hypoxia and especially the two hypoxia-inducible key regulators HIF-1α and HIF-2α have been established to be of importance in neuroblastoma progression and migration [63,64]. HIF-1α and HIF-2α have also been discussed to have a direct effect on the differentiation of neuroblasts [65,66,67]. It has, for example, been shown that hypoxia led to a de-differentiation of neuroblastoma cells that persisted for about 24 h after re-oxygenation [68]. The same group demonstrated that once neuroblastoma cells become hypoxic, they express a more immature and neural crest-like phenotype and, thus, contribute to malignancy [69,70].

An important factor which is upregulated by hypoxia in neuroblastoma and several other tumor tissues is the water channel AQP1 [21,71,72] (Huo et al., accepted January 2021). In the present case, APQ1 is expressed in the patient’s primary tumor as well as in the liver metastases. It has the capacity to transport water and plays a role in cell migration and adhesion [71]. We have previously shown that AQP1 expression leads to an increased migratory behavior of neuroblastoma cells through its upregulation under hypoxic conditions (Huo et al., published February 2021, *Front Cell Dev Biol*.). Furthermore, we have generated evidence that leads us to hypothesize that during a hypoxic window, cells are transforming towards an undifferentiated, migratory phenotype with an increased AQP1 expression [21]. Therefore, the hypoxia-dependent differentiation status of the cells could be a crucial factor in tumor development and progression of neuroblastoma.

As a possible factor in the development of CDH, hypoxia is not broadly discussed in the literature. One study on several hypoxia-inducible factors including HIF-1α and HIF-2α showed a lower expression of VEGF (vascular endothelial growth factor) mRNA in CDH patients in the alveolar stage [73]. The authors hypothesize that this might play a role in the pathophysiology of CDH. As hypoxia is closely interlinked with NMYC expression as well as being associated with other congenital malformations such as intestinal atresia, future research should take hypoxia into account as a possible cause.

## 5. Conclusions

It is clear that the underlying processes leading to congenital malformation and neonatal tumors are not yet thoroughly understood. Induction by toxins, cell differentiation and the influence of retinoic acid and NMYC as well as of hypoxia on the development of either disease entity represent only some aspects. It is highly interesting that although the disease entities appear different at first glance, some of the proposed pathophysiological aspects of both malformations are overlapping. Especially disturbances of the retinoic acid pathway and NMYC expression can influence and disrupt cell differentiation in either disease. Due to the rarity of both diseases, interdisciplinary efforts and multi-center studies are needed to investigate the reasons for congenital malformations and their interlinkage with neonatal tumor disease.

## Figures and Tables

**Table 1:** Analysis of the biopsies. The results of all biopsies led to the diagnosis of a favorable stage IV-S neuroblastoma.

Biopsy	Location	Tissue	Immunohistochemical	FISH	MGMT-STP27	Hughes Classification	Evans Classification	Evaluation
1	Thoracically herniated mass	Liver metastasis	Synaptophysin + Tyrosinhydroxylase + NB84 +	NMYC not amplified	Unmethylated	Grade 3		Poorly differentiated, stromal-poor neuroblastoma
2	Retroperitoneal	Primary tumor	Synaptophysin + Chromogranin A + Neurofilament +	NMYC not amplified			Stage 4-S	poorly differentiated, stromal-poor neuroblastoma
3	Retroperitoneal	Primary tumor	Chromogranin A +	NMYC not amplified			Stage 4-S	Poorly differentiated, stromal-poor neuroblastoma

Note: FISH: Fluorescence in situ hybridization; MGMT: O6-methylguanin-DNA-methyltransferase.

## Data Availability

All data are presented in the manuscript.
